# Beginning Restorative Activities Very Early: A Quality Improvement Project to Advance ABCDEF Bundle Practice in a Pediatric Oncology Intensive Care Unit

**DOI:** 10.3390/pediatric18040099

**Published:** 2026-07-22

**Authors:** Elizabeth Christian, Sarah Williams, Sara Tyson Husband, Amanda Brown, Mohammad Sabobeh, Sarah Schwartzberg, Eliza Hendrix, Sherry Locket, Deni Trone, Jennifer Featherston, Shankari Kalyanasundaram, Shilpa Gorantla, Maham Alam, Zhongheng Cai, Haitao Pan, Saad Ghafoor

**Affiliations:** 1 Division of General Surgery, Department of Pediatric Trauma, Le Bonheur Children’s Hospital, Memphis, TN 38103, USA; eseewer@uthsc.edu; 2 Center of Advanced Practice, Department of Nursing, St. Jude Children’s Research Hospital, Memphis, TN 38105, USA; sarah.williams2@stjude.org (S.W.); amanda.brown@stjude.org (A.B.); 3 Department of Nursing, St. Jude Children’s Research Hospital, 262 Danny Thomas Place, Memphis, TN 38105, USA; sara.husband@stjude.org; 4 Department of Pediatric Critical Care, University of Tennessee Health Science Center, Memphis, TN 38163, USA; msabobeh@health.southalabama.edu; 5 Department of Rehabilitation Services, St. Jude Children’s Research Hospital, 262 Danny Thomas Place, Memphis, TN 38105, USA; sarah.schwartzberg@stjude.org (S.S.); eliza.hendrix@stjude.org (E.H.); sherry.lockett@stjude.org (S.L.); 6 Department of Pharmacy and Pharmaceutical Sciences, St. Jude Children’s Research Hospital, 262 Danny Thomas Place, Memphis, TN 38105, USA; deni.trone@stjude.org (D.T.); maham.alam@stjude.org (M.A.); 7 Department of Information Services, St. Jude Children’s Research Hospital, 262 Danny Thomas Place, Memphis, TN 38105, USA; jennifer.featherston@stjude.org (J.F.); shankari.kalyanasundaram@stjude.org (S.K.); 8 Office of Quality and Patient Safety, St. Jude Children’s Research Hospital, 262 Danny Thomas Place, Memphis, TN 38105, USA; shilpa.gorantla@stjude.org; 9 Department of Biostatistics, St. Jude Children’s Research Hospital, 262 Danny Thomas Place, Memphis, TN 38105, USA; zhongheng.cai@stjude.org (Z.C.); haitao.pan@stjude.org (H.P.); 10 Division of Critical Care Medicine, Department of Pediatric Medicine, St. Jude Children’s Research Hospital, 262 Danny Thomas Place, Memphis, TN 38105, USA

**Keywords:** pediatric intensive care unit, quality improvement, early mobility, delirium

## Abstract

**Background/Objectives:** Children with cancer admitted to the pediatric intensive care unit (PICU) are at increased risk for post-intensive care syndrome (PICS-p) due to prolonged immobility, deep sedation, and severe illness. The ABCDEF bundle offers a framework for enhancing ICU care and patient recovery, but implementing all components in pediatric oncology patients is challenging. This study assesses the development and implementation of the BRAVE (Beginning Restorative Activities Very Early) initiative, specifically BRAVE2, to integrate the comprehensive ABCDEF bundle and a nurse-led mobility program, in collaboration with rehabilitation specialists, within a pediatric oncology intensive care unit. **Methods:** BRAVE2 was a quality improvement project conducted in a single pediatric ICU from 2022 to 2023. We analyzed ICU data to assess patient demographics, frequency of physical and occupational therapy (PT/OT) consultations, time to initial mobilization, and delirium screening rates (CAPD score of 9 or higher) for patients with ICU stays over 48 h. BRAVE2 addressed all elements of the ABCDEF bundle, including regular pain assessments, evaluation of spontaneous breathing readiness, sedation adjustments, delirium screening, early mobilization, and family engagement. Outcomes were monitored using statistical process control methods. **Results:** Of 140 patients, 117 (84%) remained in the ICU for more than 48 h. The delirium screening rate was 15.5%, consistently below the target of 30%. PT/OT consultations within 72 h occurred in 80.7% of patients, and early mobilization in 49.7% of patients, both below the 80% goal. However, 90.4% of patients with tracked mobility were able to ambulate during their ICU stay. No mobility-related safety incidents were reported. **Conclusions:** Rolling out a full ICU liberation plan in a pediatric oncology ICU is possible, and implementing a comprehensive one is feasible and sustainable despite challenges. Although therapist-led early mobilization did not meet targets, incorporating nurse-led mobility strategies and routine delirium screening has established a scalable model to enhance ICU care and support long-term recovery for these patients.

## 1. Introduction

The landscape of pediatric oncology has changed substantially with important advances in treatment options, such as hematopoietic cell transplantation, leading to improved survival outcomes. This shift has led to a focus on reducing long-term health problems and improving the quality of life for survivors of childhood cancer. However, this vulnerable group still faces a high risk of severe illness, with up to 40% of patients requiring admission to the intensive care unit (ICU) for complications related to disease or treatment, such as sepsis, respiratory failure, thrombotic microangiopathy, sinusoidal obstruction syndrome, and multiorgan dysfunction syndrome [1,2]. Paradoxically, the treatments that keep patients alive in the ICU, such as immobilization, deep sedation, and mechanical ventilation, can increase the risk of pediatric post-intensive care syndrome (PICS-p). This syndrome includes new or worsening impairments in physical, cognitive, emotional, and social domains that can persist long after ICU discharge [3,4].

Children with underlying oncologic diseases are particularly vulnerable to PICS-p. They face a synergistic combination of risks, including critical illness myopathy and neuropathy worsened by corticosteroids, neurocognitive effects from chemotherapeutic agents, and a severe catabolic state caused by both malignancy and critical illness [5,6]. The core goal of ICU liberation is to prevent patients from losing their dignity and self-worth while preserving, as much as possible, the life they knew before their critical illness [7]. Our group previously reported on the safety and feasibility of the BRAVE (Beginning Restorative Activities Very Early) early mobility initiative, demonstrating that a structured, multidisciplinary approach can increase the number of rehabilitation consults and early mobilization in this high-acuity population [8]. The BRAVE initiative is based on the evidence-based ABCDEF bundle, an integrated approach to patient care that enhances outcomes across a broad range of critical illnesses. It is a comprehensive framework for ICU liberation that includes *assessment* and management of pain, both spontaneous awakening trials and spontaneous *breathing* trials (SBTs), *choice* of light sedation, *delirium* assessment and management, *early* mobility and exercise (through physical therapy [PT] and occupational therapy [OT] consults), and *family* engagement and empowerment [9,10].

The present study describes the evolution and maturation of the BRAVE initiative in a new era marked by systemic challenges, including the COVID-19 pandemic, a transition to a new electronic health record (EHR) system (Epic), and substantial staff turnover. We build on our initial work by implementing the entire ABCDEF bundle, moving beyond a focus on delirium and early PT/OT-driven mobility to incorporate and evaluate all six elements (BRAVE2). Additionally, we introduce a novel nurse-based mobility strategy that extends throughout the ICU stay, ensuring that rehabilitation is a continuous practice rather than limited to the first 72 h.

This study aimed to evaluate the implementation of a comprehensive ABCDEF bundle strategy in a pediatric oncology ICU and to describe process measures for delirium screening, mobilization practices, rehabilitation consultations, and nurse-led mobility implementation.

## 2. Materials and Methods

BRAVE2 is a single-center, retrospective quality improvement (QI) study that builds on the initial BRAVE study (hereafter referred to as BRAVE1), which established early mobility protocols in the pediatric ICU (PICU) at St. Jude Children’s Research Hospital from 2019 to 2020 [8]. BRAVE2 extends the evaluation to a 2022–2023 cohort and examines ICU encounter-level trends in PT/OT consultation, patient mobilization, and compliance with Cornell Assessment of Pediatric Delirium (CAPD) screening and outcomes.

BRAVE2 builds on the previously published BRAVE1 initiative, which established the foundation for early mobility practices in our pediatric oncology ICU and demonstrated the feasibility and safety of therapist-led mobilization. The current project focused on expanding ICU liberation practices to all ABCDEF bundle components and incorporating a nurse-driven mobility strategy. While formal pre-post comparisons were not performed, the previously reported BRAVE1 metrics provide important context for interpreting the program’s evolution [8].

The primary research objectives of BRAVE2 were (1) to characterize the PICU patient population, (2) to determine the rate of PT/OT consultation and mobilization within 72 h of ICU admission, and (3) to evaluate the monthly rate of positive delirium screens (CAPD ≥ 9) among patients with an ICU length of stay (LOS) > 48 h.

Specific, Measurable, Achievable, Relevant, and Time-bound (SMART) aim targets were established before project implementation. These included a positive delirium screening rate of ≤30%, PT/OT consultation rate ≥80%, and mobilization rate ≥80%.

This manuscript was prepared in accordance with the SQUIRE 2.0 reporting guidelines for quality improvement studies.

### 2.1. Health Care Personnel Training

Successful implementation of BRAVE2 required multidisciplinary education and ongoing competency development. Training was provided to bedside nurses, advanced practice providers, attending physicians, physical therapists, occupational therapists, respiratory therapists, pharmacists, and quality improvement personnel. Educational sessions focused on ICU liberation principles, use of the Cornell Assessment of Pediatric Delirium (CAPD), BRAVE mobility levels, extubation readiness processes, sedation optimization, mobility safety procedures, and family engagement strategies. New staff received onboarding education and reinforcement occurred through multidisciplinary rounds, quality meetings, and periodic workflow reviews.

### 2.2. Optimizing Pain Assessment and Management

Effective pain control is fundamental to ICU liberation, as untreated pain frequently leads to escalating sedation, prolonged mechanical ventilation, and delayed mobilization [7,9,11]. To support this bundle element, we standardized age-appropriate pain assessment using validated scales: the FLACC scale for nonverbal children and numeric rating scales for verbal children.

Pain scores were documented at regular intervals and reviewed during multidisciplinary rounds. Severe or persistent pain prompted early involvement of pain management providers, enabling the timely implementation of multimodal analgesia strategies. This approach aimed to address discomfort proactively rather than increasing continuous sedative infusions in response to agitation, consistent with pediatric sedation best practices [11,12]. Additionally, structured pain assessment facilitated targeted sedation decisions and supported safe participation in SBTs and the nurse-led mobility initiative.

### 2.3. Optimizing Spontaneous Breathing Trials

Extubation readiness trials (ERTs) play a crucial role in pediatric critical care, enhancing outcomes for young patients by accurately determining when extubations can occur. By carefully evaluating spontaneous breathing and airway protection, ERTs provide an evidence-based approach to minimize the risk of extubation failure [13,14,15]. Incorporating ERTs into clinical practice can lead to shorter durations of mechanical ventilation and fewer ventilator-associated complications [16,17,18]. Therefore, ERTs are an important strategy for improving patient outcomes and ensuring the efficient use of resources in PICUs.

We established a standardized protocol for daily ERTs. Ventilator liberation is now a fixed agenda item during twice-daily multidisciplinary rounds. During these rounds, the care team, which included dedicated respiratory therapists (RTs), collaboratively evaluated each patient’s suitability for ERT, considering sedation levels, restraint use, cognitive status, and overall clinical trajectory. Patient eligibility for an ERT was determined according to our strict ICU ERT protocol (Figure S1).

### 2.4. Optimizing the Choice of Sedation

Sedation practices were intentionally aligned with ICU liberation principles, emphasizing lighter sedation and the preferential use of non-benzodiazepine regimens when clinically feasible [7,9]. Sedation depth was monitored using the Richmond Agitation Sedation Scale and incorporated into twice-daily multidisciplinary rounds [19].

Dexmedetomidine was recommended as the preferred first-line sedative infusion, given its favorable delirium profile and ability to facilitate SBTs and early mobilization [20,21]. Ketamine emerged as one of the most used sedative infusions during the study period, reflecting its dual analgesic-sedative properties and hemodynamic stability in medically complex oncology patients [22]. Benzodiazepines were reserved for specific clinical indications, consistent with evolving evidence demonstrating increased delirium risk associated with their use [9,20].

### 2.5. Optimizing Delirium Assessment and Management

**Figure 1:**
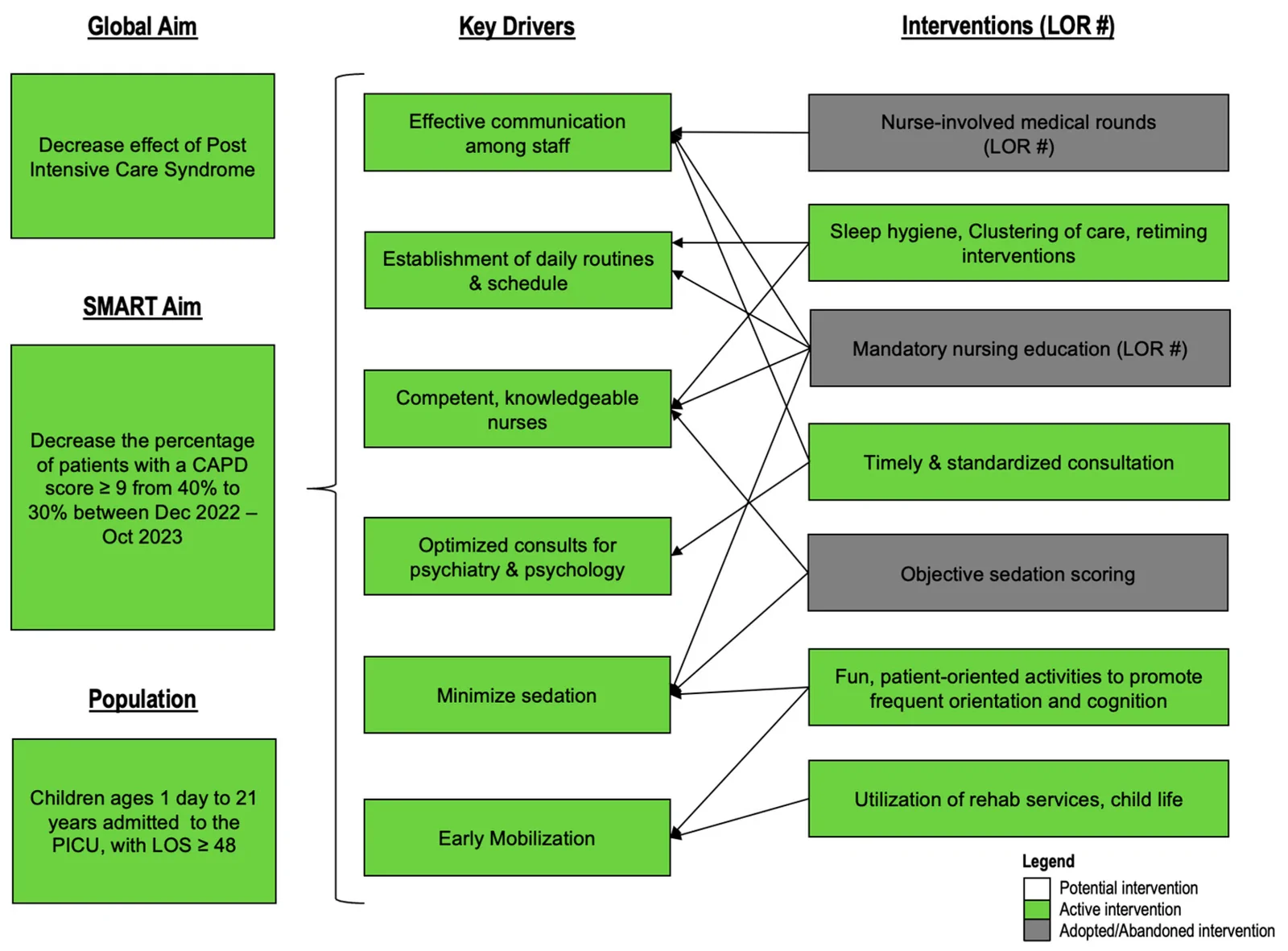
Key driver diagram targeting delirium. CAPD, Cornell Assessment of Pediatric Delirium; PICU, pediatric intensive care unit; LOR, level of reliability; LOR # = Level of Reliability Number, e.g., LOR 1; LOS, length of stay.

Delirium is highly prevalent in critically ill pediatric oncology patients and represents a major barrier to safe mobilization, ventilator liberation, and recovery [3,23]. To strengthen this element of the ABCDEF bundle, we standardized twice-daily delirium screening using the CAPD, a validated pediatric tool [24]. The SMART aim was to decrease the percentage of patients with a CAPD score ≥ 9 among those admitted to the ICU for more than 24 h. (Figure 1).

Delirium screening results were incorporated into multidisciplinary rounds, prompting discussion of sedation depth, sleep hygiene, environmental modifications, and the need for psychiatry or psychosocial service involvement. Systematic screening reframed delirium as a modifiable clinical variable rather than an expected consequence of critical illness.

### 2.6. Optimizing Early Mobility and Nurse-Based BRAVE Levels

Early mobility within the oncology PICU faces unique challenges, including thrombocytopenia, fluctuating clinical status, and profound fatigue. Pediatric early mobility programs have demonstrated safety and feasibility when implemented within structured, multidisciplinary frameworks [8,23,25].

**Figure 2:**
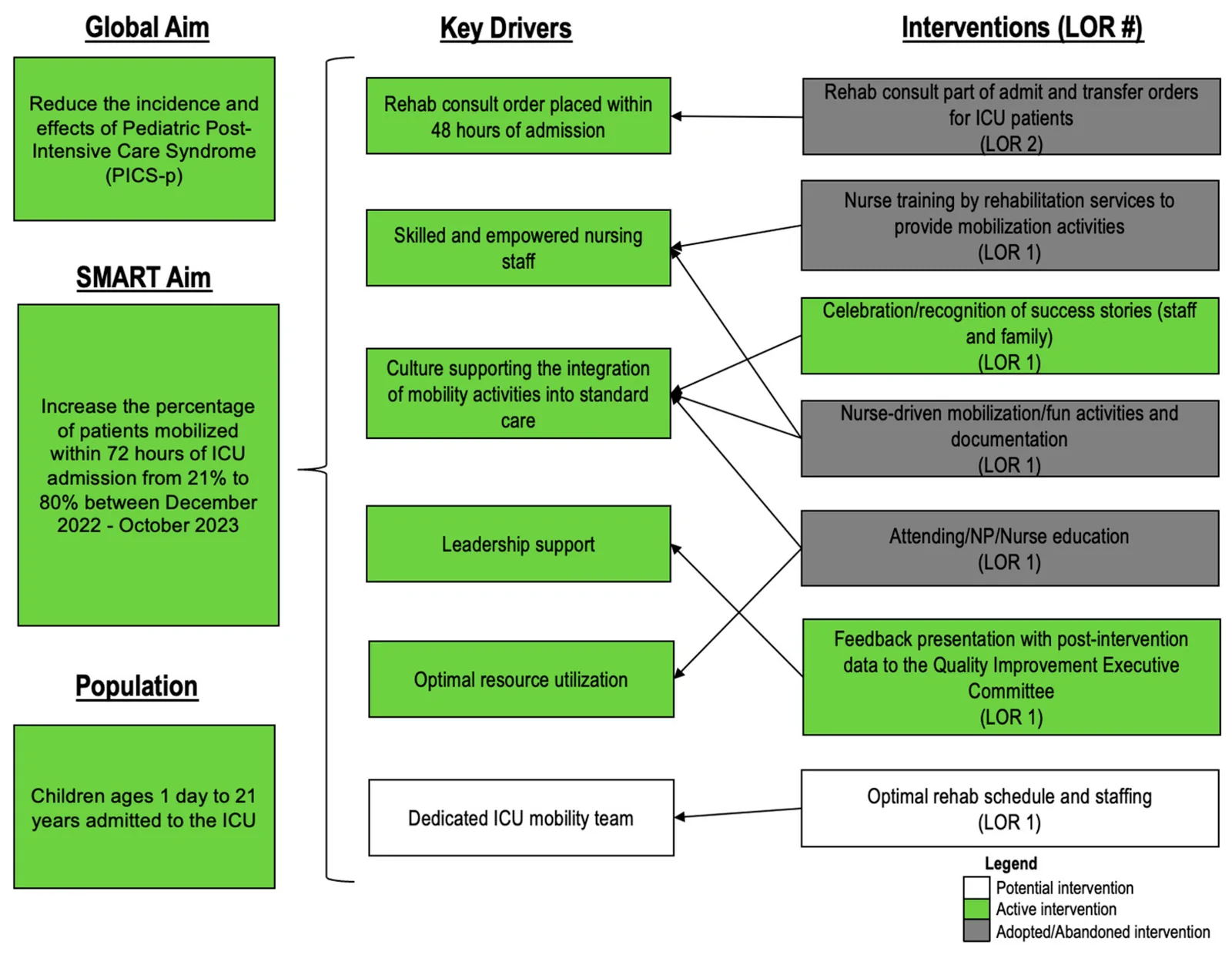
Key driver diagram targeting early mobility of patients in the intensive care unit (PICU). LOR, level of reliability; LOR # = Level of Reliability Number, e.g., LOR 1.

BRAVE1 focused on PT/OT consult placement and mobilization within the first 72 h of ICU admission, whereas this second phase focused on progression of the mobility model. In BRAVE2, mobility was reframed from a therapist-driven intervention to a continuous, nurse-driven responsibility integrated throughout the entire ICU stay. The SMART aim was to increase the percentage of patients mobilized within 72 h of ICU admission to ≥80% between December 2022 and October 2023 (Figure 2).

Standardized BRAVE mobility levels (Table 1) were assigned during rounds and documented daily by bedside nursing staff, guiding progression from in-bed activities to ambulation.

### 2.7. Optimizing Family Engagement

Family engagement is one of the least-studied and most difficult-to-measure components when assessing ICU liberation. Limited literature exists on family engagement in critical care settings, with restrictions noted regarding optimal frequency and variable engagement levels due to external factors across centers [26]. During the implementation of BRAVE1, we discovered that gaining our families’ trust was imperative to the program’s success. Using three strategies, we sought to gain and maintain this trust by providing early introduction to the ICU liberation program and its importance, daily family-centered rounds, and encouraging active participation of families in patient care.

When patients are first admitted to the PICU, families are given an educational packet that introduces the concept of ICU liberation and details each part of the ABCDEF bundle (Figure S2). Admission to a PICU is often stressful and unexpected, leaving families and patients feeling overwhelmed. By introducing this education during the initial admission, families can learn how we are working to promote comfort and healing, and to maintain the most normal routine possible for their child. This approach ensures that patients and families have time to read and understand the information and ask questions as they arise.

Family-centered rounds are another method to build trust with our patients and families. When families are able to witness the medical team’s discussion, they report greater understanding and trust in the final medical decision [27]. These rounds occur twice daily, with a multidisciplinary team meeting directly outside or sometimes inside the patient’s room. The patient’s ICU course is presented by the medical team, and both patients and families are invited to provide input for the daily plan of care. We believe this approach gives families a sense of empowerment and control during a difficult time and encourages them to remain engaged in their child’s care and treatment.

Family participation in patient care has been shown to be crucial to implementing and sustaining ICU liberation for pediatric patients. Most of the patients admitted to our PICU are either newly diagnosed or well-established. Understandably, there is heightened fear of the unknown among the families of newly diagnosed oncology patients. By fostering relationships between parents and their child’s medical team and rehabilitation specialists, parents gain confidence to continue bonding with and caring for their child while adapting to a new routine. Furthermore, this confidence is maintained in the families of our established patients. PICU admission often interrupts the carefully crafted routine that these families have developed to care for their child, and some aspects of care must be adapted to provide safety for the child. ICU liberation and family participation in medical discussions enable families to remain active in their child’s care.

Family engagement activities were monitored through routine implementation audits, verification of the distribution of educational materials, and documentation of family-centered rounds within clinical workflows. However, no formal quantitative measure of family engagement, family satisfaction, or caregiver experience was collected during the study period. Therefore, this component is reported qualitatively and represents an important area for future study.

To facilitate implementation and standardization of care processes, an operational workflow diagram of the BRAVE2 ICU liberation pathway was developed and is provided in the Supplemental Material (Figure S6).

### 2.8. Data Collection and Analysis

Time trends were examined using statistical process control methodology to support ongoing QI monitoring.

Monthly rates are displayed as proportion control charts (p-charts), with variable control limits (±3σ) calculated using the overall period proportion as the center line. No formal hypothesis testing or pre- vs. post-implementation comparison was conducted in BRAVE2. All analyses were performed using R software (version 4.4.0; R Core Team 2021, R Foundation for Statistical Computing). Continuous variables are reported as median (interquartile range, [IQR]), whereas categorical variables are reported as N (%).

Special-cause variation was defined as non-random variation suggestive of a meaningful change in process performance, such as data points outside control limits or sustained shifts and trends according to standard statistical process control rules.

## 3. Results

Patient demographics (Table 2) are summarized for the full cohort (All LOS, *N* = 140) and the LOS > 48 h subgroup (*n* = 117). Among the 140 unique patients, 117 (84%) had an ICU LOS > 48 h. The cohort was 58% male, with a median age of 8.0 years (IQR 4.0–15.2). The median ICU LOS for the full cohort was 4.1 days (IQR 2.2–9.0), increasing to 4.8 days (IQR 3.0–10.2) for the LOS > 48 h subgroup, as expected given the filtering criterion. Most admissions were urgent (96%). The largest proportion (29%) of patients were admitted to the leukemia and lymphoma service, followed by the solid tumor service (23%) and surgery service (15%), consistent with the oncology-focused patient population at St. Jude Children’s Research Hospital.

### 3.1. Spontaneous Breathing

Initial compliance with the ERT protocol and documentation during the study period was 64%, providing a strong foundation for implementing SBTs in clinical practice. We believe true adherence was likely higher; however, this baseline rate underscores the opportunity for continued improvement. To support sustained progress, we implemented monthly compliance audits and feedback sessions to reinforce performance and optimize adoption of this practice.

Our team-based approach achieved a notable safety milestone, with zero unplanned extubations during the study period. This outcome reflects our proactive, collaborative care model, in which RTs are constantly present in the ICU and at the bedside for all procedures—from bathing and transfers to imaging and interventions. The active involvement and real-time feedback of RTs optimized sedation management, reduced the risk of delirium, and enhanced patient safety, ultimately improving clinical outcomes and preventing unplanned extubations.

Daily sedation interruptions were not formally documented during the study period. Nonetheless, our protocol required a scheduled ERT at 06:00 each day for all eligible patients. Additionally, an afternoon trial was performed, at the discretion of the rounding team, for patients anticipated to undergo extubation, providing further support for the ventilator liberation process.

### 3.2. Choice of Sedation

During the study period, dexmedetomidine was the most frequently administered sedative agent (114 patients), whereas ketamine and propofol were administered in 39 and 69 patients, respectively (Table 3). These sedation patterns demonstrate increased utilization of dexmedetomidine and ketamine, with decreased reliance on continual infusions of morphine and propofol. Dexmedetomidine was the most administered sedative agent.

Intermittent bolus dosing was not consistently captured in the EHR, and therefore, total sedative exposure may be underestimated. Additionally, patient acuity and oncologic complexity likely influenced agent selection, limiting causal interpretation.

Many patients received more than one sedative agent during their ICU course; therefore, medication categories are not mutually exclusive, and cumulative patient counts exceed the total number of patients studied.

### 3.3. Delirium Assessment and Management

**Figure 3:**
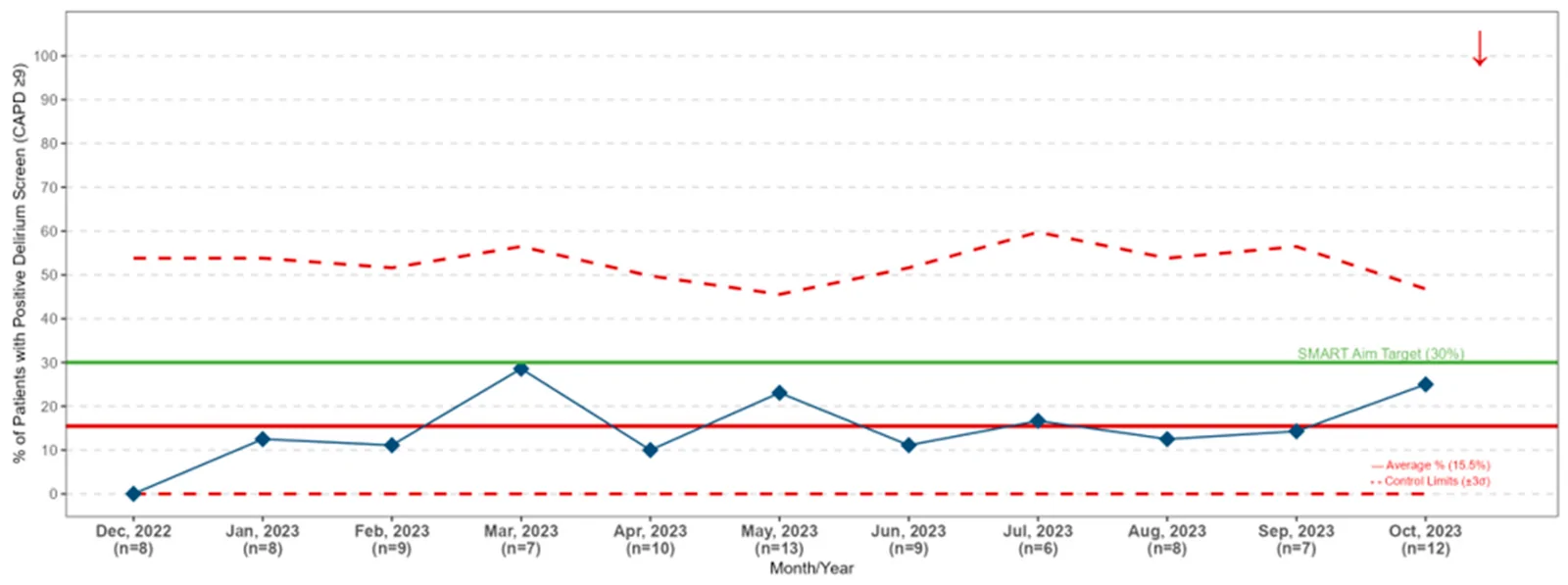
P-chart of Cornell Assessment of Pediatric Delirium (CAPD)-positive delirium screens. Blue line = monthly positive delirium screen rate (CAPD score ≥ 9) among patients with LOS > 48 h. Center line (red solid) = overall mean 15.5%; dashed red lines = ±3σ control limits; green line = 30% SMART aim target. No evidence of special-cause variation was observed, indicating process performance remained stable throughout the study period.

During the study period, 81 patients with an ICU LOS > 48 h were eligible for delirium screening, among whom 15 (19%) had at least one positive CAPD score (≥9). Monthly positive delirium screens ranged from 0% to 28.6%, with an overall rate of 15.5%, remaining consistently below the SMART target of ≤ 30% (Figure 3). All observed values were within statistical control limits, indicating a stable process without evidence of special-cause variation.

The relatively low rate of positive delirium screens coincided with the implementation of standardized delirium screening and multidisciplinary review processes. However, because there was no control group or pre-post comparison, a causal relationship between BRAVE2 implementation and delirium outcomes cannot be established.

Although screening compliance and positive-delirium screen rates were captured, delirium severity trajectories and time to resolution remain under evaluation. Pharmacologic management strategies were not standardized early in the study period, which limited the assessment of the treatment effect.

### 3.4. Early Mobility and Nurse-Driven BRAVE Levels

**Figure 4:**
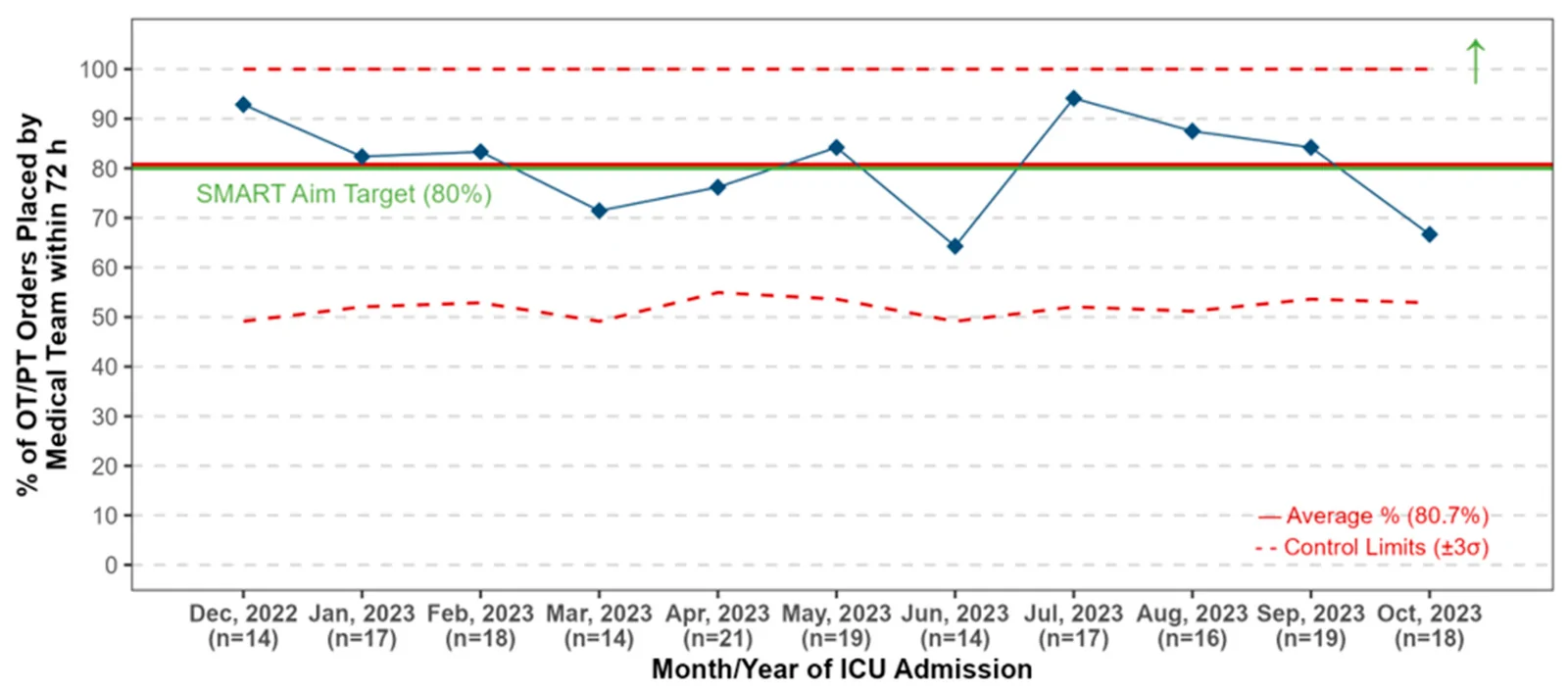
Monthly PT/OT consult rates within 72 h of ICU admission (p-chart). Center line (red solid) = overall mean 80.7%; dashed red lines = ±3σ control limits; green line = 80% SMART aim target; blue line = monthly PT/OT consult rates. No evidence of special-cause variation was observed, indicating process performance remained stable throughout the study period.

Early mobility was reframed from a therapist-driven intervention to a continuous, nurse-based responsibility in BRAVE2. The SMART aim targets were to increase the rates of PT/OT consultation and mobilization to ≥80%. Overall, the mean PT/OT consultation rate within 72 h of ICU admission was 80.7% (Figure 4).

**Figure 5:**
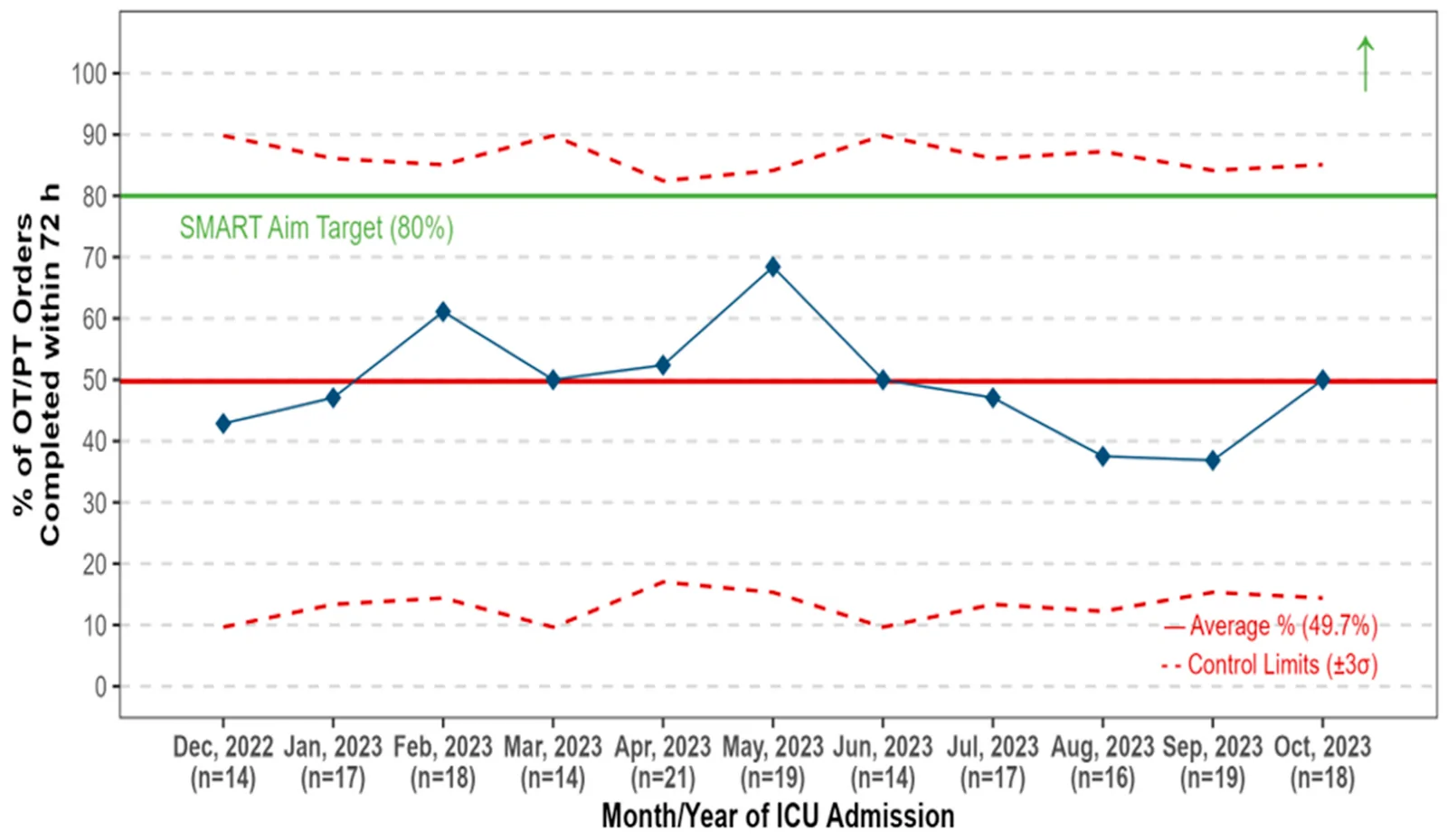
Monthly patient mobilization rate within 72 h of ICU admission (p-chart). Center line (red solid) = overall mean 49.7%; dashed red lines = ±3σ control limits; green line = 80% SMART aim target; blue line = monthly patient mobilization rate. No evidence of special-cause variation was observed, indicating process performance remained stable throughout the study period.

However, only 49.7% of consultations were fully completed within 72 h. Among consultations not completed within this timeframe, 39.3% were at least attempted by rehabilitation staff. When completed and attempted consultations were combined, 69.5% of patients had rehabilitation services addressed within 72 h of ICU admission. (Figure 5).

A summary of denominator calculations is provided in Table 4. Overall, (69.5%) were addressed within 72 h, but operational challenges affect completion. Medical instability was the main reason for deferral in 17 cases (46%), followed by scheduling conflicts in 8 cases (22%). Provider concern and caregiver refusal each appeared in 4 cases (11%), while patient refusal and other reasons were less common (Table 5). Despite positive trends, performance fell short of institutional targets. These results show that although early rehab is active, consistent delivery to every eligible patient remains challenging due to patient- and operational-related barriers.

Nursing documentation data included 887 records across 125 unique patients and 874 patient-days, allowing for longitudinal evaluation of mobility practices. Standardized BRAVE mobility levels (Table 1) were assigned during rounds and documented daily by bedside nursing staff, guiding progression from in-bed activities to ambulation (Table 6).

Within the first 72 h of ICU admission, mobility appropriate to the assigned BRAVE level occurred in 10.4% of Level 1 patient-days, 77.3% of Level 2 patient-days, and 91.0% of Level 3 patient-days (Table S1). These rates increased over the course of the ICU stay to 14.7% for Level 1, 84.4% for Level 2, and 91.5% for Level 3 (Table S1).

At the patient level, 113 of 125 patients (90.4%) achieved ambulation (Level 3) at some point during their ICU stay, whereas 12 patients (9.6%) had no documented mobilization (Table S2). When examined longitudinally, mobility was documented on a median of 50% of ICU days (IQR 30–66.7%), suggesting substantial variability in mobility exposure across the cohort (Table S3).

Importantly, this expansion of nurse-initiated mobility occurred without any documented adverse events, including unplanned extubation, central line dislodgement, or patient or staff injury (Table 4). A limitation of this analysis is that mobility classification relied on keyword-based extraction from free-text nursing documentation, which may underestimate lower-level activities such as passive range of motion. Additionally, variability in documentation across providers and incomplete capture of all patients (125 of 140 total) may affect the generalizability of these findings.

## 4. Discussion

This quality improvement project demonstrates the feasibility and sustainability of implementing a comprehensive ABCDEF bundle strategy within a pediatric oncology intensive care unit. The study primarily evaluates process measures related to bundle implementation and does not directly assess patient-centered clinical outcomes, staff experience, or family satisfaction.

Similar ICU liberation and early mobilization initiatives have been reported across pediatric and adult critical care settings. In pediatrics, the PICU Up! quality improvement program demonstrated that a structured, interdisciplinary early mobilization strategy could be safely implemented in critically ill children, resulting in increased rehabilitation consultations, greater mobility activity, and no mobilization-related adverse events [25]. Subsequent multicenter studies, including the PARK-PICU investigations conducted in North America, Europe, Canada, and Brazil, confirmed that mobilization of critically ill children is feasible and generally safe, with low rates of adverse events despite substantial variation in practice patterns and persistent barriers such as mechanical ventilation, oversedation, and limited rehabilitation resources [28,29,30,31,32]. Broader pediatric ICU liberation efforts have also gained momentum. An international survey found that complete implementation of all ABCDEF bundle components occurred in only 9% of PICUs, with early mobility and delirium monitoring representing the least frequently adopted elements [33]. More recently, the Pediatric ICU Liberation Collaborative demonstrated that increased utilization of the ABCDEF bundle was associated with significantly lower mortality, supporting the potential value of comprehensive liberation strategies in children [34].

Current SCCM guidelines recommend routine delirium screening and standardized early mobility protocols, although the overall quality of evidence remains limited [35].

In adult critical care, implementation of the ABCDEF bundle has been associated with improved survival, reduced delirium, shorter duration of mechanical ventilation, and other favorable patient-centered outcomes, with a clear dose–response relationship between adherence to the bundle and clinical benefit [9,36,37].

However, pediatric oncology patients represent a uniquely vulnerable population because of treatment-related immunosuppression, thrombocytopenia, neuromuscular weakness, and prolonged hospitalizations. Consequently, evidence supporting the safety, feasibility, and effectiveness of comprehensive ICU liberation strategies, particularly in pediatric oncology critical care, remains sparse, underscoring the need for further evaluation in this specialized population.

Importantly, our results highlight both successes and ongoing gaps in bundle implementation. Early PT/OT consultation achieved the target of over 80%, reaching 80.7% within 72 h of ICU admission. However, mobilization stayed below the goal at 49.7%. Performance was stable over time, with no evidence of special-cause variation. These findings suggest that staffing limitations, competing clinical priorities, and the complexity of pediatric oncology patients continue to restrict early therapist-driven mobilization, even with established protocols.

In contrast, delirium outcomes were favorable. The overall rate of positive delirium screens (CAPD ≥ 9) was 15.5%. This was consistently below the predefined target of 30%. This likely reflects the successful integration of standardized delirium screening into the daily workflow and the increased recognition of delirium as a modifiable clinical condition. While causality cannot be established, these findings suggest coordinated efforts in sedation management, environmental optimization, and multidisciplinary care helped maintain low rates of positive delirium screens.

The successful implementation of a nurse-based mobility strategy, operationalized through standardized BRAVE levels, marks a crucial shift in practice. It changes rehabilitation from a specialist-led, time-limited intervention to a fundamental nursing duty woven into every shift. This approach ensures mobilization is a continuous process throughout the ICU stay, regardless of PT/OT availability. Nurse documentation data indicated that most patients achieved high levels of mobility, with about 90% ambulating at some point during their ICU stay. Mobility was also documented on about half of ICU days, providing a longitudinal measure of mobility exposure beyond the 72 h window. This change represents a meaningful evolution from early mobility to sustained, integrated mobility across the entire ICU course.

The complete bundle generates a synergistic effect. Structured spontaneous breathing trials and a preference for lighter, non-benzodiazepine sedation supported safer participation in mobility activities. Systematic delirium screening helped identify and address major mobility barriers. Sedation practices like using dexmedetomidine and ketamine align with ICU liberation principles. These aim to minimize the need for deep sedation and facilitate ventilator liberation and mobility. Increased family involvement also allowed caregivers to actively support their child’s recovery and mobilization. This integrated approach is especially important in the pediatric oncology population, where fluctuating conditions, fatigue, and specific vulnerabilities like thrombocytopenia or neuropathic pain can delay recovery.

Notably, expansion of the nurse-based mobility initiative and integration of the bundle occurred without any documented adverse events. These included unplanned extubations, central line dislodgements, or patient or staff injuries. Although no mobility-related adverse events were documented during the study period, this finding reflects the experience of this single-center quality improvement initiative and should not be interpreted as evidence that such events cannot occur in other clinical settings, patient populations, or larger implementation efforts. Ongoing safety monitoring remains an essential component of early rehabilitation programs.

Despite these successes, challenges remain. Nurse-to-patient ratios, competing clinical priorities, and the complexity of medically fragile pediatric patients remain major barriers to optimal adherence. Limits in structured data capture, especially for pain assessment, sedation dosing, and mobility intensity, restricted our ability to fully quantify certain bundle elements. Measuring long-term impacts on functional outcomes, neurocognitive performance, and quality of life after discharge remains important, yet logistically challenging.

A formal cost-effectiveness analysis was not performed. Consequently, the financial implications of implementing a comprehensive ICU liberation strategy, including personnel resources, training requirements, and operational costs, could not be evaluated.

Future studies should assess the economic impact and sustainability of these programs. Our future initiatives will address these gaps through targeted, system-level strategies. Priorities include developing an ‘ABCDEF Bundle Compliance’ metric to improve performance tracking. We will use EHR-based decision support tools to standardize care and optimize documentation workflows, which should reduce administrative burden and improve data accuracy. Other initiatives include establishing a multidisciplinary PICS-p follow-up program to evaluate long-term outcomes and guide early interventions. Given ongoing barriers to therapist-led mobility, adopting dedicated ICU PT/OT staffing models is a critical next step. This will improve timeliness, reduce inefficiencies, and better align rehabilitation delivery with patient readiness.

In summary, these findings underscore the durability and evolution of ICU liberation practices in a pediatric oncology ICU. Substantial progress has been achieved, notably through nurse-driven mobility and sustained reductions in delirium. Persistent gaps in the timing of early mobilization highlight the need for ongoing system-level innovation. Strengthening alignment between rehabilitation intent and execution will be essential for consistent, high-quality ICU liberation and better outcomes in this vulnerable population.

## 5. Limitations

Several limitations should be considered when interpreting these findings. The retrospective quality improvement design limits the ability to determine whether observed changes were attributable to BRAVE2 implementation itself or to concurrent institutional practice changes occurring during the same period.

While the frequency of pain assessment documentation was captured, qualitative pain severity trajectories and response-to-intervention timing were not uniformly recorded. Additionally, bolus analgesic administration was not reliably extractable from the electronic health record, limiting granular analysis of total opioid exposure. These limitations restricted our ability to fully evaluate the relationship between pain-management practices and ICU liberation outcomes.

Although no mobility-related adverse events were observed during the study period, the sample size was relatively small, and the absence of observed events should not be interpreted as evidence that complications cannot occur. Nevertheless, our findings are consistent with prior reports suggesting that structured mobility programs can be implemented safely when supported by multidisciplinary teams and standardized protocols.

Our institution possesses specialized pediatric oncology expertise, established rehabilitation services, and multidisciplinary intensive care unit (ICU) resources that may not be available at other centers. Facilities with lower nurse-to-patient ratios, fewer rehabilitation personnel, or limited pediatric oncology experience may encounter different implementation challenges. These factors could restrict the generalizability of the findings. This study acknowledges several limitations. First, the retrospective quality improvement design precludes causal inference and constrains the ability to distinguish intervention effects from concurrent institutional changes. Second, the study was conducted in a single specialized pediatric oncology ICU, which may limit its applicability to other settings. Third, several bundle components, including family engagement, were assessed qualitatively rather than quantitatively. Fourth, structured data collection for pain management, mobility intensity, and cumulative sedative exposure was incomplete. Fifth, clinical outcomes such as mortality rates, duration of mechanical ventilation, post-intensive care syndrome, long-term functional recovery, staff satisfaction, and family experience were not evaluated. Lastly, although no mobility-related adverse events were observed, the sample size was relatively modest, and rare complications might not have been identified.

While the implementation took place in a pediatric oncology ICU with dedicated rehabilitation resources, several aspects of the BRAVE2 program could be adapted for centers with different staffing models. These include standardized mobility assessments, integrating mobility goals into daily interdisciplinary rounds, staff training on early rehabilitation, and improved communication within care teams. Such strategies can support the adoption of mobility programs even in settings with fewer specialized rehabilitation staff, although local modifications would be needed. Additionally, routines like extubation readiness trials, delirium screening and management, sleep prioritization, family involvement and empowerment, and optimized use of sedatives and opioids can be implemented in most units. Even with limited resources, mobility efforts involving nurses and parents, without the need for physical therapists or specialized equipment, can be largely achieved. Future multi-center implementation studies are needed to determine which program elements have the greatest impact across diverse practice settings.

## 6. Conclusions

This quality improvement initiative demonstrates the successful implementation of an early mobilization program in a pediatric oncology ICU and supports practices that may contribute to improved patient experiences and recovery, warranting further study of their impact on clinical and long-term outcomes. Although early therapist-driven mobilization remained below target, integration of key ABCDEF bundle elements into routine nursing practice, particularly mobility and delirium assessment, provided a scalable model that expanded rehabilitation opportunities beyond therapist availability. By embedding ICU liberation principles into unit culture and leveraging the multidisciplinary team, the project established a durable framework that supports both immediate clinical outcomes and the long-term recovery of children with cancer and blood disorders. These findings add to the limited literature on comprehensive ABCDEF bundle implementation in pediatric oncology critical care. Future studies should evaluate patient- and family-centered outcomes, staff experience, long-term functional recovery, and post-intensive care syndrome to better define the overall impact of ICU liberation strategies in this unique population.

## Figures and Tables

**Table 1:** Standardized BRAVE mobility levels. ROM, range of motion; OOB, out of bed.

BRAVE Level	Activity Keywords (Case-Insensitive)
Level 1	sitting up in bed, sit up, dangle, dangling, range of motion, ROM, repositioning, splint, level 1 activities
Level 2	Level 1 keywords + chair, OOB, out of bed, out-of-bed, sitting up/OOB, level 2 activities
Level 3	Level 2 keywords + ambulate, ambulation, walk, level 3 activities

**Table 2:** Patient demographics. LOS, length of stay; IQR, interquartile range.

Patient Characteristic	All LOS (N = 140)	LOS > 48 h (n = 117)
Age, years—median (IQR)	8.0 (4.0–15.2)	8.0 (4.0–15.0)
Sex, N (%)		
Male	81 (58%)	62 (53%)
Female	59 (42%)	55 (47%)
ICU LOS, days—median (IQR)	4.1 (2.2–9.0)	4.8 (3.0–10.2)
Admission type, N (%)		
Urgent	135 (96%)	114 (97%)
Elective	3 (2%)	2 (2%)
Emergency	1 (1%)	0 (0%)
Service, N (%)		
Leukemia	41 (29%)	35 (30%)
Solid tumor	32 (23%)	28 (24%)
Surgery	21 (15%)	12 (10%)
Intensive care	16 (11%)	16 (14%)
Transplant	13 (9%)	12 (10%)
Hematology	11 (8%)	8 (7%)
Neuro-oncology	6 (4%)	6 (5%)

**Table 3:** Sedation drug doses are summarized as the median (IQR) of the patient-level mean daily dose.

Sedation Agent	N	Median Dose (IQR)
Ketamine (mg)	39	50 (25.8–75.0)
Dexmedetomidine (µg/kg/h)	114	0.65 (0.45–0.90)
Propofol (mg)	69	55 (30.5–85.0)

**Table 4:** Rehab consults.

OT/PT ICU Consults	
Orders placed by medical team within 48 h	(80.7%)
OT/PT Orders completed within 72 h	(49.7%)
OT/PT orders attempted within 72 h (N = 94-consults not completed)	(39.3%)
Total Consults completed or addressed within 72 h	(69.5%)

**Table 5:** Rehab Mobility outcome measures.

Deferral Reasons for OT/PT Orders Not Being Completed Within 72 h	
Medical Status	17 (46%)
Schedule conflict/procedure/test	8 (22%)
Provider concern	4 (11%)
Caregiver refusal	4 (11%)
Patient refusal	2 (5%)
Other	2 (5%)
Lack of staff	0 (0%)

**Table 6:** Nursing Mobility outcome measures.

Nurse Led Mobility-Outcome Measure	n (%)
Activity type (Nursing records, N = 887 rows)	
In-bed activity (ROM, repositioning, splinting, sitting)	842 (95%)
Out-of-bed activity (ambulation, standing, transfer, chair)	275 (31%)
Level of assistance (N = 839 valid rows)	
Completely dependent	469 (56%)
Assistive person required	308 (37%)
Independent	38 (5%)
Assistive equipment	17 (2%)
Assistive equipment and person	7 (1%)
Deferral reason (N = 189 records)	
Schedule conflict/procedure/test	4 (2%)
Provider concern	2 (1%)
Patient refusal	0 (0%)
Caregiver refusal	0 (0%)
Lack of staff	0 (0%)
Adverse events	
Unplanned extubation	0 (0%)
Unintentional removal of central venous catheter	0 (0%)
Staff injury	0 (0%)
Patient injury	0 (0%)

## Data Availability

The original contributions presented in this study are included in the article. Further inquiries can be directed to the corresponding author.

## Supplementary Materials

The following supporting information can be downloaded at: https://www.mdpi.com/article/10.3390/pediatric18040099/s1, Figure S1: ERT Protocol; Figure S2: Bedside Physical Therapy to Promote Upright Tolerance and Functional Recovery; Figure S3: Bedside Music Therapy Promoting Sensorimotor Engagement in a Pediatric Patient; Figure S4: Therapist-Assisted Functional Mobility Training During Critical Illness Recovery; Figure S5: Tricycle-Assisted Mobility Training in a Pediatric Inpatient Setting; Figure S6: BRAVE2 ICU Liberation Pathway; File S1: ICU Liberation—A Plan of Care for Your Child’s Comfort and Recovery (packet); Table S1: Daily Mobilization Rate Appropriate to BRAVE Level (Patient-Day Level); Table S2: Highest Mobilization Level Achieved During ICU Stay (Patient Level); Table S3: Proportion of ICU Days with Any Mobilization Documented.

## Abbreviations

LOR	Level of Reliability
SMART	Specific, Measurable, Achievable, Relevant, Time-bound
FLACC	Face, Legs, Activity, Cry, Consolability
SBT	Spontaneous Breathing Trial
BRAVE	Beginning Restorative Activities Very Early
CAPD	Cornell Assessment of Pediatric Delirium
ERT	Extubation readiness trial
EHR	Electronic health record
ICU	Intensive care unit
IQR	Interquartile range
LOS	Length of stay
OT	Occupational therapist
PICS-p	Pediatric post-intensive care syndrome
PICU	Pediatric intensive care unit
PT	Physical therapist
QI	Quality improvement
RT	Respiratory therapist
