# Supplementary material for: Beginning Restorative Activities Very Early: A Quality Improvement Project to Advance ABCDEF Bundle Practice in a Pediatric Oncology Intensive Care Unit
Source: Pediatr Rep. 2026 Jul 22;18(4):99. doi: 10.3390/pediatric18040099 (PMC13516045; doi:10.3390/pediatric18040099)
Supplement: Supplementary file 1 [file pediatrrep-18-00099-s001.zip › pediatrrep-4403089-Supplementary Figures and Tables.pdf]

Table S1: Daily Mobilization Rate Appropriate to BRAVE Level (Patient-Day Level)

BRAVE Level	Within 72h N/Patient-Days (%)	Overall ICU Stay N/Patient-Days (%)
Level 1	5/48 (10.4%)	29/197 (14.7%)
Level 2	17/22 (77.3%)	141/167 (84.4%)
Level 3	132/145 (91.0%)	335/366 (91.5%)

Presents the proportion of patient-days on which nursing-documented activity was appropriate to the assigned BRAVE level, reported separately for the first 72 hours of ICU admission and for the overall ICU stay. Level 2 and Level 3 patients demonstrated high rates of appropriate daily mobilization, both within the first 72 hours (77.3% and 91.0%, respectively) and across the full ICU stay (84.4% and 91.5%). Level 1 patients showed substantially lower rates (10.4% within 72h; 14.7% overall). This likely reflects the nature of Level 1 activities — in-bed exercises such as repositioning and passive ROM — which may be performed routinely but are documented less consistently in the Activities field compared to higher-level mobility tasks.

Table S2: Highest Mobilization Level Achieved During ICU Stay (Patient Level)

Highest Activity Level Achieved	N (%)
Level 3: Ambulation	113 (90.4%)
Level 2: OOB to Chair	0 (0.0%)
Level 1: In-bed activities	0 (0.0%)
No mobilization documented	12 (9.6%)

Summarizes the highest level of mobility activity documented for each of the 125 patients in the nursing dataset across their entire ICU course. Many patients (90.4%) achieved ambulation — the highest mobility level — at some point during their ICU stay. No patients were classified at Level 1 or Level 2 as their highest level, meaning all patients with any documented mobility activity ultimately progressed to ambulation. The 12 patients with no mobilization documented (9.6%) may reflect either clinical contraindications or gaps in nursing documentation. These findings warrant confirmation with the PI regarding whether the keyword definitions capture all intended Level 1 and Level 2 activities.

Table S3: Proportion of ICU Days with Any Mobilization Documented

Metric	Value
Median % ICU days with any mobilization (IQR)	50% (IQR: 30%–66.7%)

Presents the distribution of the proportion of ICU days on which any nursing-documented mobilization activity was recorded, computed at the patient level. The median patient had nursing-documented mobility on approximately half of their ICU days (50%; IQR 30%–66.7%). The interquartile range reflects meaningful variability across patients: some patients were mobilized on fewer than one-third of their ICU days, while others achieved mobilization on more than two-thirds. This metric provides a longitudinal view of mobility exposure that complements the 72-hour snapshot and the overall stay rate reported above.

St. Jude - Confidential

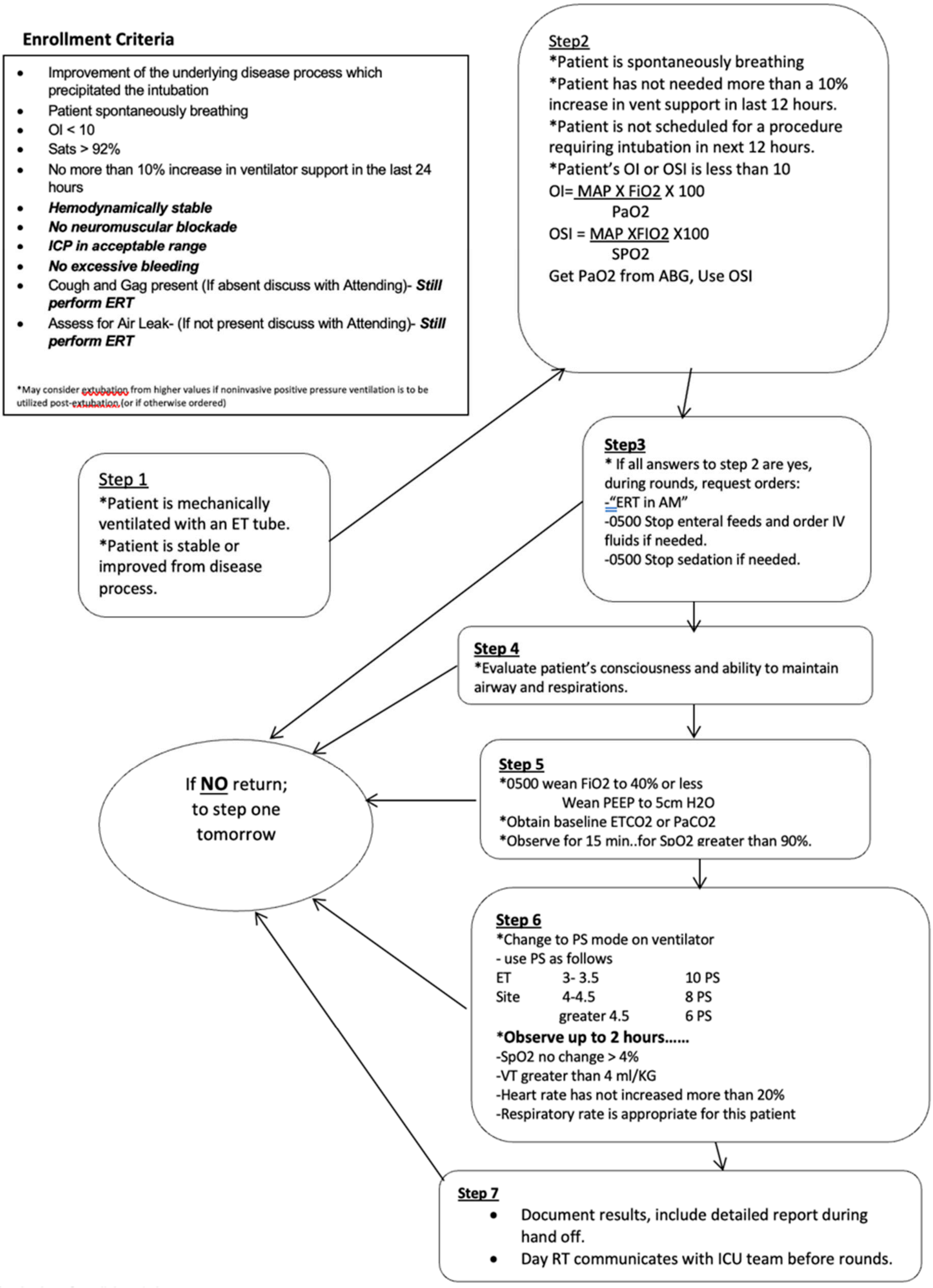

Figure S1. ERT Protocol.

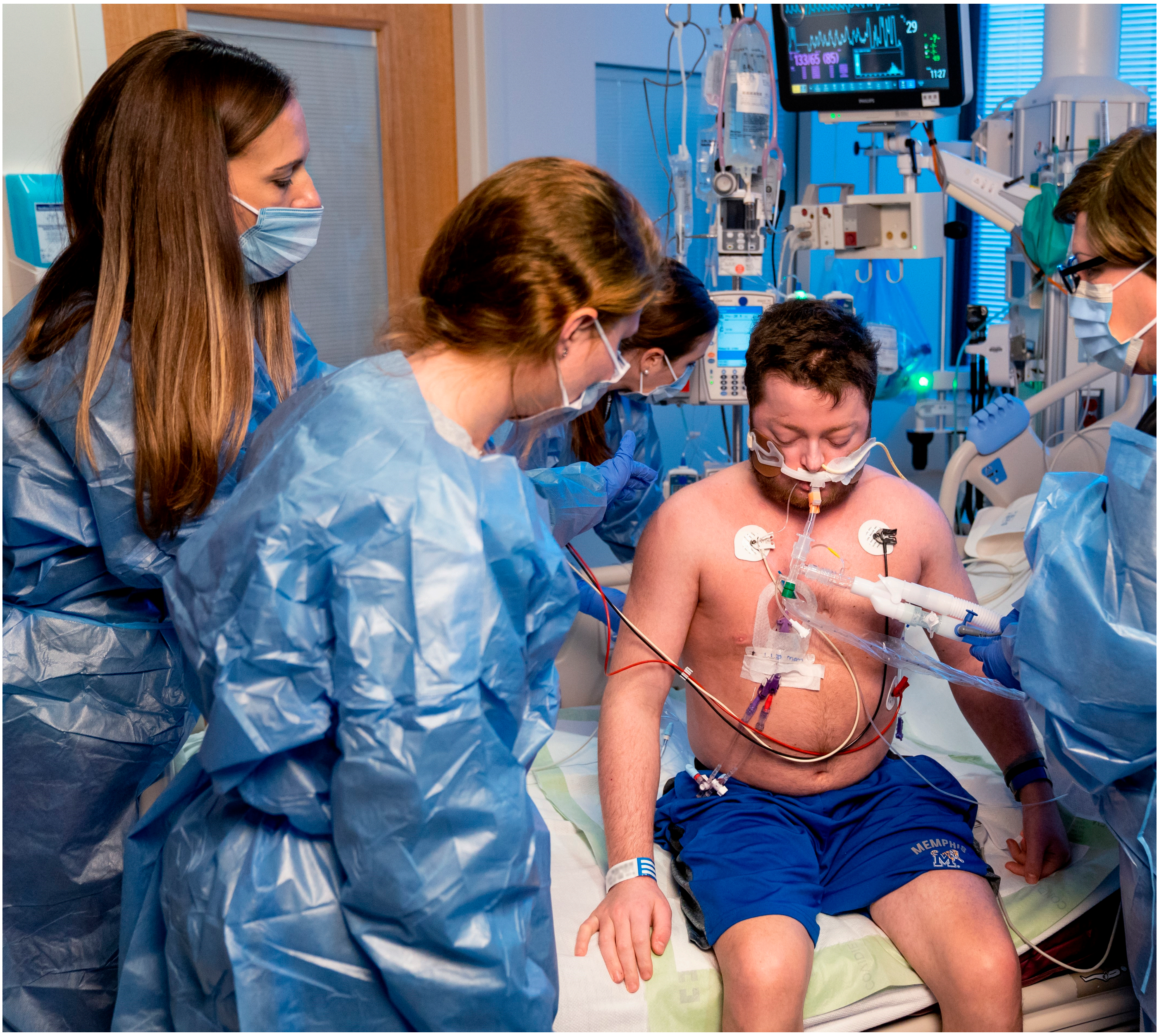

Figure S2. Bedside Physical Therapy to Promote Upright Tolerance and Functional Recovery. Written informed consent has been obtained from the patients (guardians) to publish this figure. If you have any questions, please contact correspondence author.

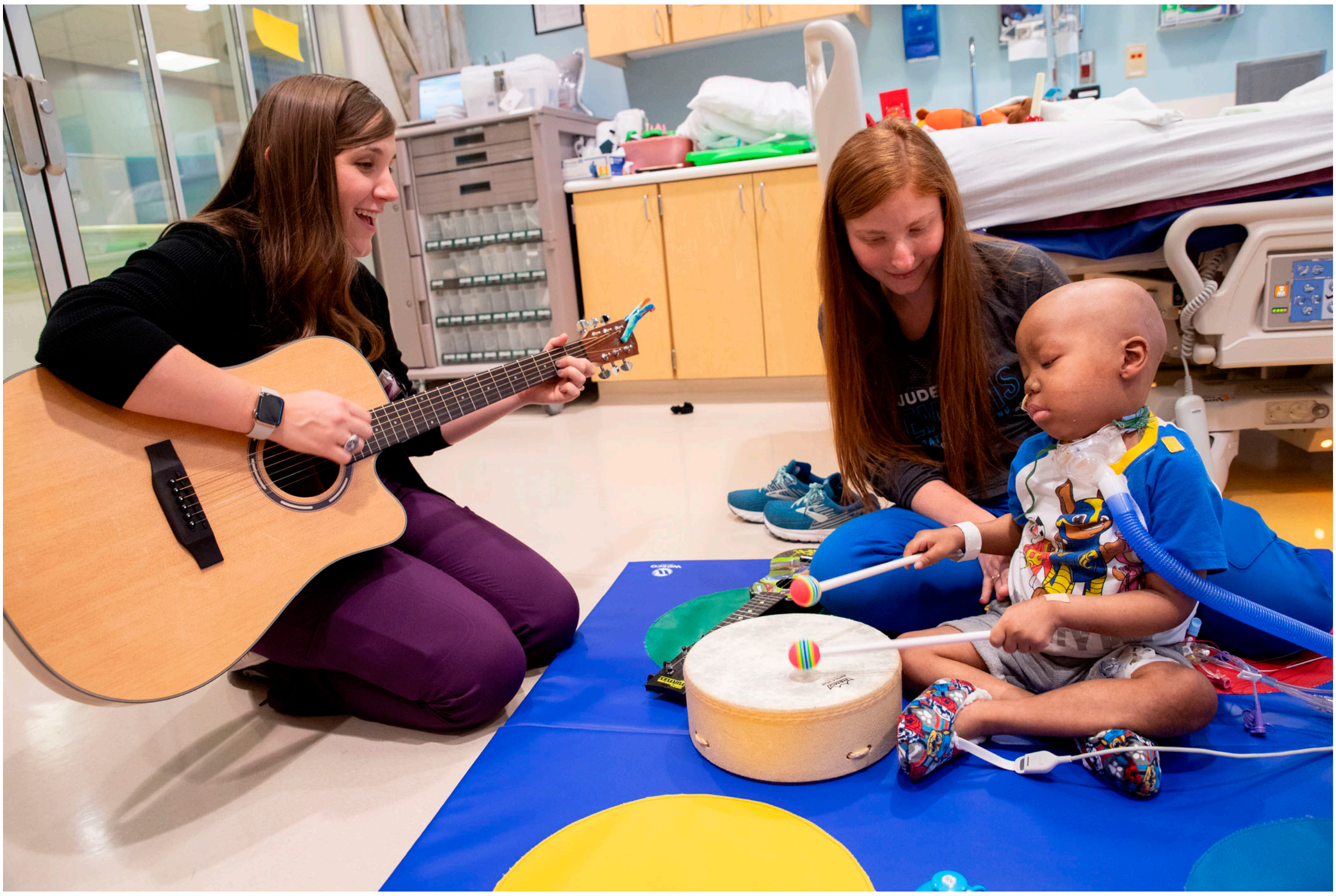

Figure S3: Bedside Music Therapy Promoting Sensorimotor Engagement in a Pediatric Patient. Written informed consent has been obtained from the patients (guardians) to publish this figure. If you have any questions, please contact correspondence author.

St. Jude - Confidential

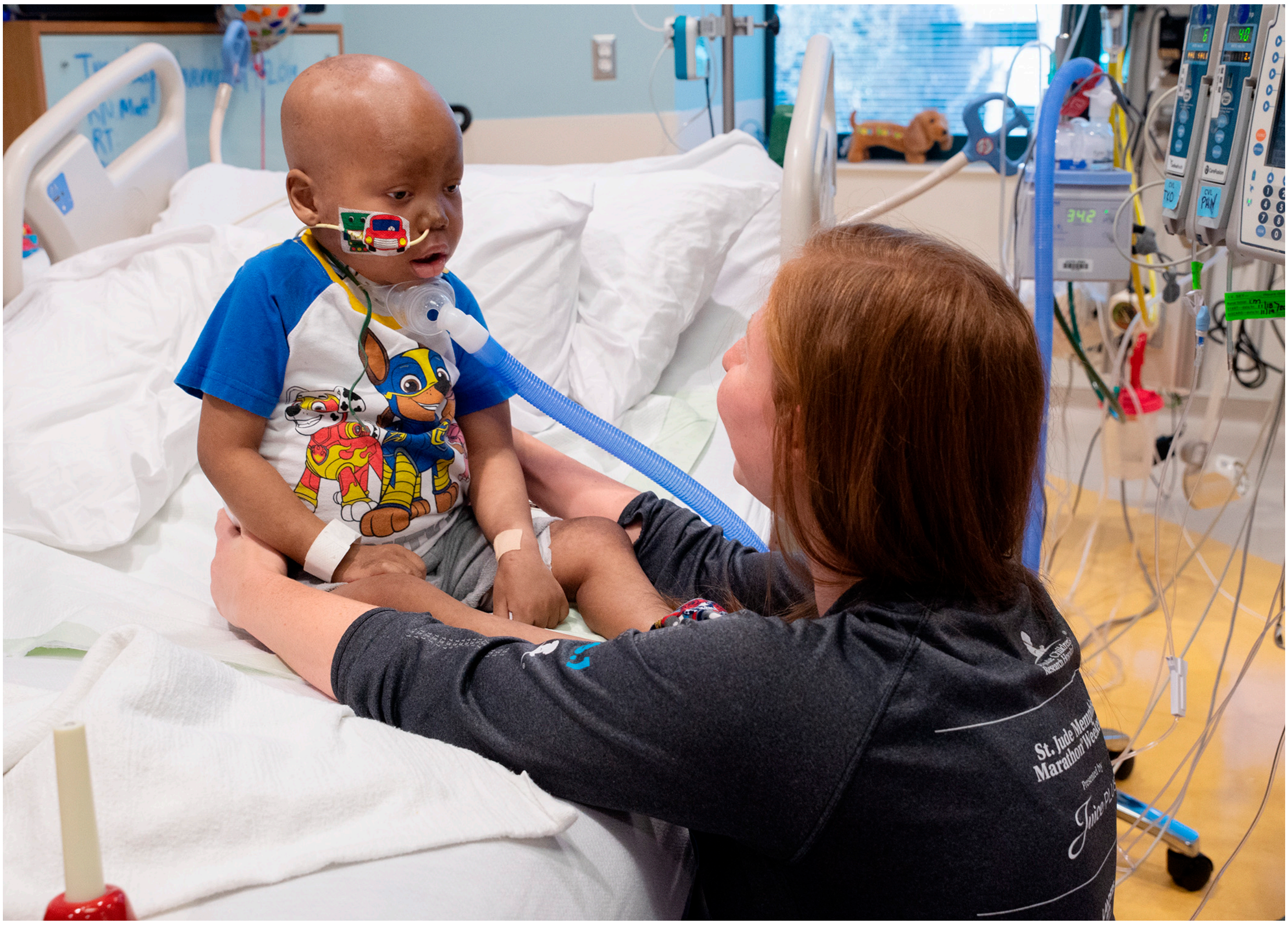

Figure S4: Therapist-Assisted Functional Mobility Training During Critical Illness Recovery. Written informed consent has been obtained from the patients (guardians) to publish this figure. If you have any questions, please contact correspondence author.

St. Jude - Confidential

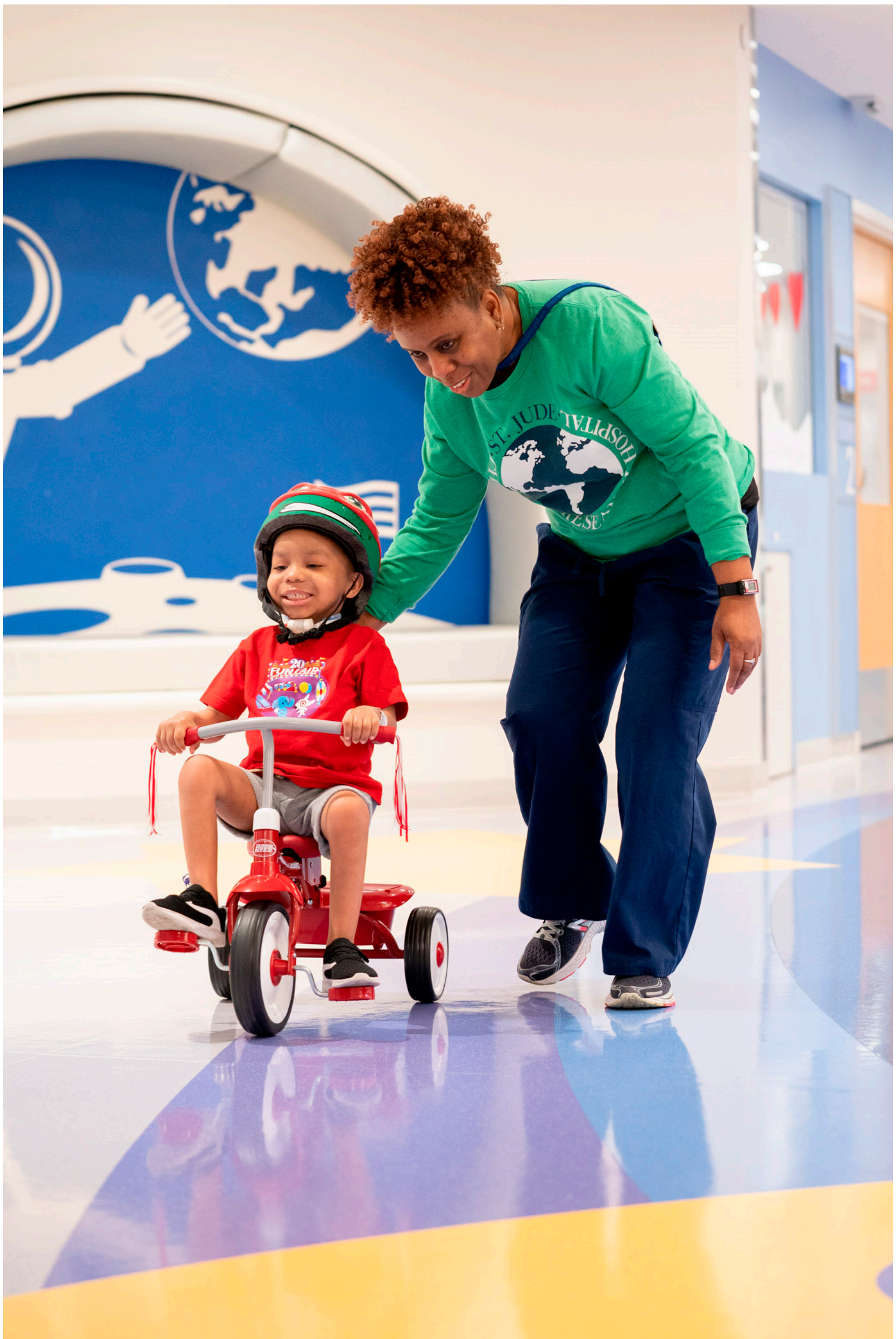

Figure S5: Tricycle-Assisted Mobility Training in a Pediatric Inpatient Setting. Written informed consent has been obtained from the patients (guardians) to publish this figure. If you have any questions, please contact correspondence author.

PICU Admission

↓

Pain Assessment and Management (A)

↓

Extubation Readiness Evaluation (B)

↓

Sedation Optimization (C)

↓

Delirium Screening and Management (D)

↓

Daily BRAVE Mobility Assignment (E)

↓

Family Engagement and Participation (F)

↓

Daily Reassessment During Multidisciplinary Rounds

↓

Continuous Mobility Progression Throughout ICU Stay

Figure S6. BRAVE2 ICU Liberation Pathway.

St. Jude - Confidential
