# Supplementary material for: Beginning Restorative Activities Very Early: A Quality Improvement Project to Advance ABCDEF Bundle Practice in a Pediatric Oncology Intensive Care Unit
Source: Pediatr Rep. 2026 Jul 22;18(4):99. doi: 10.3390/pediatric18040099 (PMC13516045; doi:10.3390/pediatric18040099)
Supplement: Supplementary file 1 [file pediatrrep-18-00099-s001.zip › pediatrrep-4403089-Supplementary File.pdf]

File S1: ICU Liberation – A Plan of Care for Your Child's Comfort and Recovery (packet).

ICU Liberation: A Plan of Care for Your Child's Comfort and Recovery

The Intensive Care Unit (ICU) team is committed to providing your child with the best care possible. We use a series of steps known as the ICU Liberation Bundle. This plan of care helps promote your child's comfort and healing during their ICU stay. Our goal is to maintain as normal a routine as possible for your child.

A: Assess and Manage Pain

Managing your child's pain is a top priority. The ICU team will use several strategies to keep your child comfortable.

Pain assessment: The team will assess your child's pain using special pain scales designed for children. The team will also look for signs of pain, such as facial expressions, body movements, and vocalizations (sounds). Pain assessment will be done every 4 hours or more often if needed. This lets us know if your child has pain and how severe it is. The team will use this information to develop a comprehensive pain management plan.

Pain management with medicines: One part of the plan is to use pain medicines such as acetaminophen, ibuprofen, or opioid medicines. The ICU team will prescribe pain medicines based on your child's specific needs.

Pain management without medicines: The ICU team will also recommend other pain management strategies. These may include techniques such as warm blankets, cold packs, positioning, massage, music therapy, aromatherapy, and distraction.

The goal of pain management is to find the right combination of treatments to manage pain safely and effectively. The team will closely monitor your child's response to pain treatments and make changes as needed. Good pain control is crucial. Unmanaged pain can increase stress and delay healing. If you think your child might be in pain, let a care team member know.

B: Breathing Trials

Many children in the ICU need to be on a ventilator (breathing machine) and sedation medicines to support their recovery. However, use of these interventions for a long time can cause other problems.

The ICU team will conduct "trials" to help your child wean off ventilator support and sedation as soon as possible.

First, the team may perform a Spontaneous Awakening Trial (SAT). This is where sedation medicines are briefly paused or reduced to see if your child can wake up and interact.

Next, the team will conduct an Extubation Readiness Trial (ERT). If the child passes 1 or 2 of the ERTs 12 hours apart, then a Spontaneous Breathing Trial (SBT) may be conducted. In this step, the team will temporarily disconnect your child from the ventilator and place them on a self-inflating anesthesia bag to see if they can breathe on their own.

The team will closely monitor your child's vital signs and level of alertness during these trials.

Passing these trials shows that your child is ready to have their sedation and

breathing support reduced or stopped. This allows your child to regain control of their own breathing and become more alert and engaged in their recovery process.

C: Choosing the Right Medications

Your child will likely need a combination of medicines to keep them comfortable in the ICU. This may include medicines to prevent pain, anxiety, and agitation. However, certain types of medicines can have side effects, such as withdrawal symptoms or confusion.

The ICU team will avoid medicines that are more likely to cause dependency and withdrawal issues. Instead, the team may use medicines with the least harmful effects along with non-medication therapies like music or massage to promote comfort and reduce anxiety. They will adjust the dose of medicine and wean your child off as they improve.

The ICU team will carefully select medicines based on your child's medical needs. The goal is to keep your child comfortable while also helping them participate in their care and avoid long-term complications.

D: Delirium Prevention and Management

Delirium is a serious condition characterized by sudden changes in mental status. These changes may include confusion, disorientation, restlessness, unusual behavior, agitation, hallucinations, and changes in attention or alertness.

Delirium is very common in the ICU, especially in children. If not managed, delirium can have negative effects such as increased distress, risk of injury, longer hospital stays, and long-term cognitive problems.

To help prevent delirium, the team will take proactive measures, such as:

Maintaining normal sleep-wake cycles

Reducing environmental stressors such as noise and bright lights

Encouraging involvement of familiar people, objects, and activities

Treating any underlying medical issues contributing to delirium

The ICU team will closely monitor your child for any signs of delirium using specialized assessment tools. If delirium does develop, interventions may include medication changes, reorientation techniques to remind your child where they are, and encouraging movement and mobility. The goal is to prevent it from happening and reduce the intensity if it does happen.

E: Early Mobility and Exercise

A lack of mobility or movement can cause problems such as muscle weakness, joint stiffness, decreased fitness, and worse outcomes. To avoid these issues, the ICU team will work to get your child moving within 12 hours of admission, according to what's medically appropriate.

Early mobility includes personalized activity and exercise to get your child moving safely. This may start with simple exercises in bed, like moving their arms and legs or gently sitting up. As your child's condition improves, the team will progress to more advanced movements like standing and walking.

The ICU team will work with physical therapists and occupational therapists to develop an individualized exercise plan that considers your child's specific medical needs, abilities, and goals. They will encourage you and other family members to actively participate in these mobility sessions so your child will regain strength and function.

Early mobility can significantly improve your child's short-term recovery, reduce

their length of stay in the ICU, and prevent long-term physical disabilities.

F: Family Engagement

The ICU team understands that having a child in the ICU is a stressful and overwhelming experience for families. The team is committed to actively involving patients and families in care and decision-making.

First, the team will provide comprehensive education about your child's condition, the treatments being used, and the ICU plan of care. This will help you better understand the reasoning behind the care plan and feel empowered to participate.

Next, the team will encourage you to take an active, hands-on role in supporting your child's recovery. This may include providing comfort measures, assisting with mobility exercises, and communicating your child's needs to the medical staff. You are an integral part of the care team.

You will also be included in regular care conferences, where the full ICU team will update you on your child's progress and collaborate with you on changes to the treatment plan. Your input and preferences are valued and will be included in decision-making.

A strong partnership between the family and the medical team helps ensure your child receives the most personalized, supportive care possible during this critical time.

G: Good Sleep Hygiene

Quality sleep is essential for healing and recovery, especially for patients who are critically ill. However, the ICU environment can often disrupt normal sleep-wake cycles. With your help, the ICU team will use strategies to promote healthy sleep for your child. These include:

Controlling light and noise levels in the room, especially at night

Limiting nighttime disruptions for care activities, such as checking vital signs and giving medicines

Encouraging relaxation techniques like quiet music, massage, or guided imagery

Establishing a consistent bedtime routine, such as dimming lights, reading stories, or doing calming activities

The team may also work with you to identify and address any underlying issues impacting your child's sleep, such as pain, anxiety, or delirium. Proper sleep hygiene can have a profound impact on your child's physical and emotional healing, as well as their long-term outcomes.

Through this complete approach, we can reduce complications, enhance your child’s comfort, promote patient family involvement, and support your child’s recovery. Please let a care team member know if you have any questions.
